# Retraction Note: Sexual reproductive health service utilization and associated factors among undergraduate students of Addis Ababa University in Ethiopia

**DOI:** 10.1186/s41043-023-00388-0

**Published:** 2023-05-22

**Authors:** Bethel Mekbib, Dereje Bayissa Demissei

**Affiliations:** 1 Department of Reproductive Health, Sante Medical College, Addis Ababa, Ethiopia; 2 St. Paul’s Hospital Millennium Medical College, Addis Ababa, Ethiopia

**Retraction Note: Journal of Health, Population and Nutrition (2023) 42:21** https://doi.org/10.1186/s41043-023-00363-9

The Editor-in-Chief has retracted this article due to irregularities in the peer review process. The Editor-in-Chief therefore no longer has confidence in the results and conclusions of this article.

Author Dereje Bayissa Demissei stated that both authors disagree with the retraction.

